# SIMPEDVR: using VR in teaching pediatric emergencies to undergraduate students—a pilot study

**DOI:** 10.1007/s00431-023-05254-z

**Published:** 2023-10-16

**Authors:** Sandro Savino, Giulia Mormando, Giorgia Saia, Liviana Da Dalt, Todd P. Chang, Silvia Bressan

**Affiliations:** 1 Department of Medicine, University of Padua, Padua, Italy; 2 Department of Neurosciences, University of Padua, Padua, Italy; 3 Department of Women’s and Children’s Health, University of Padua, Padua, Italy; 4 University of Southern California, Children’s Hospital Los Angeles, Los Angeles, CA USA

**Keywords:** Pediatric, Emergency, Virtual reality, Medical education

## Abstract

The objective of this work was to provide pilot data on feasibility of using virtual reality (VR) to train undergraduate students in pediatric emergency scenarios. We staged VR sessions for a total of 45 medical and nursing students; in every session, each student managed two pediatric emergency virtual scenarios. At the end of the sessions, students completed a Technology Assessment Questionnaire to evaluate the perceived usefulness and perceived ease-of-use of their VR training experience and rated their perceived level of competence in managing the two clinical scenarios. The median perceived usefulness was 91.7/100 (interquartile range (IQR) 80.6–100), while the median perceived ease-of-use was 77.8/100 (IQR 63.9–88.9). The perceived level of competence increased from 2 (IQR 1–3) to 4 (IQR 3–4) on a 5-point Likert scale, for both scenarios (*p* < 0.001, Wilcoxon test for paired samples).

*       Conclusions*: The staged VR sessions had a good perceived usefulness and resulted in an increase in the perceived level of competence. The results on the ease-of-use, however, show that an assumption that millennials and younger students can navigate with confidence VR hardware in a healthcare training setting should not be made; further work is required to ease the integration of VR into curricula.

**Table:** 

**What is Known:** *• Virtual reality (VR) is a rising simulation training methodology in Pediatric Emergency Medicine (PEM), however little experience is reported about its use for undergraduate students*	
**What is New:** *• VR PEM trainiing was found useful by undergraduate students and its use increased their perceived level of competence, although ease-of-use received lower ratings.* *• Despite the young age, an assumption that millennials and younger students can navigate with confidence VR hardware in a healthcare training setting should not be made*

**Supplementary Information:**

The online version contains supplementary material available at 10.1007/s00431-023-05254-z.

Pediatric Emergency Medicine (PEM) is not formally recognized as board-certified specialty in most European countries [1]. Trainees and physicians have expressed discomfort with pediatric emergency management [2–4], and optimal methods to increase their comfort and competence are needed. Virtual reality (VR) is a rising and common simulation training methodology used in PEM [5–9] that has increased since 2020 [10]. While it has been deployed in many areas of the world, its feasibility among European physician trainees and students for PEM is relatively unknown. The objective of our work was to provide pilot data on feasibility in an academic Italian medical school using a localized VR simulation program previously used in the USA, called ResuscitationVR (i3 Simulations, London, UK) [11]. We describe the pilot project with students, report data collected from this initial cohort, and the resultant implementation changes for the subsequent years.

In 2021, we staged nine VR sessions, involving 5 students each at the University of Padova, Italy. ResuscitationVR was provided on 5 separate Oculus Quest 2 headsets (Meta, San Jose, CA, USA). The sessions were offered to last year medical and nursing students. Students managed two pediatric virtual scenarios: an infant with status epilepticus and a child with anaphylaxis.

ResuscitationVR was initially developed to elicit a heightened psychological state [11]. Immersive audio provides sounds typical of the setting (monitor alarms, patient’s moans, the mother’s vocalizations, and intercom announcements), which adds stress and creates a realistic environment.

To help students to familiarize with the headset, a 15-min introduction on its use and a software tutorial were given beforehand. The tutorial had been developed to blind the students from the actual scenario details. We replaced all of the medical equipment, medications, and characters, and instead placed virtual rubber duckies in different locations in the emergency room to teach navigation and interactivity with the ducks using the VR controllers (Fig. 1a). It was meant to help students understand how to use the VR controllers to interact with equipment and medications in the medical scenario.

**Fig. 1:**
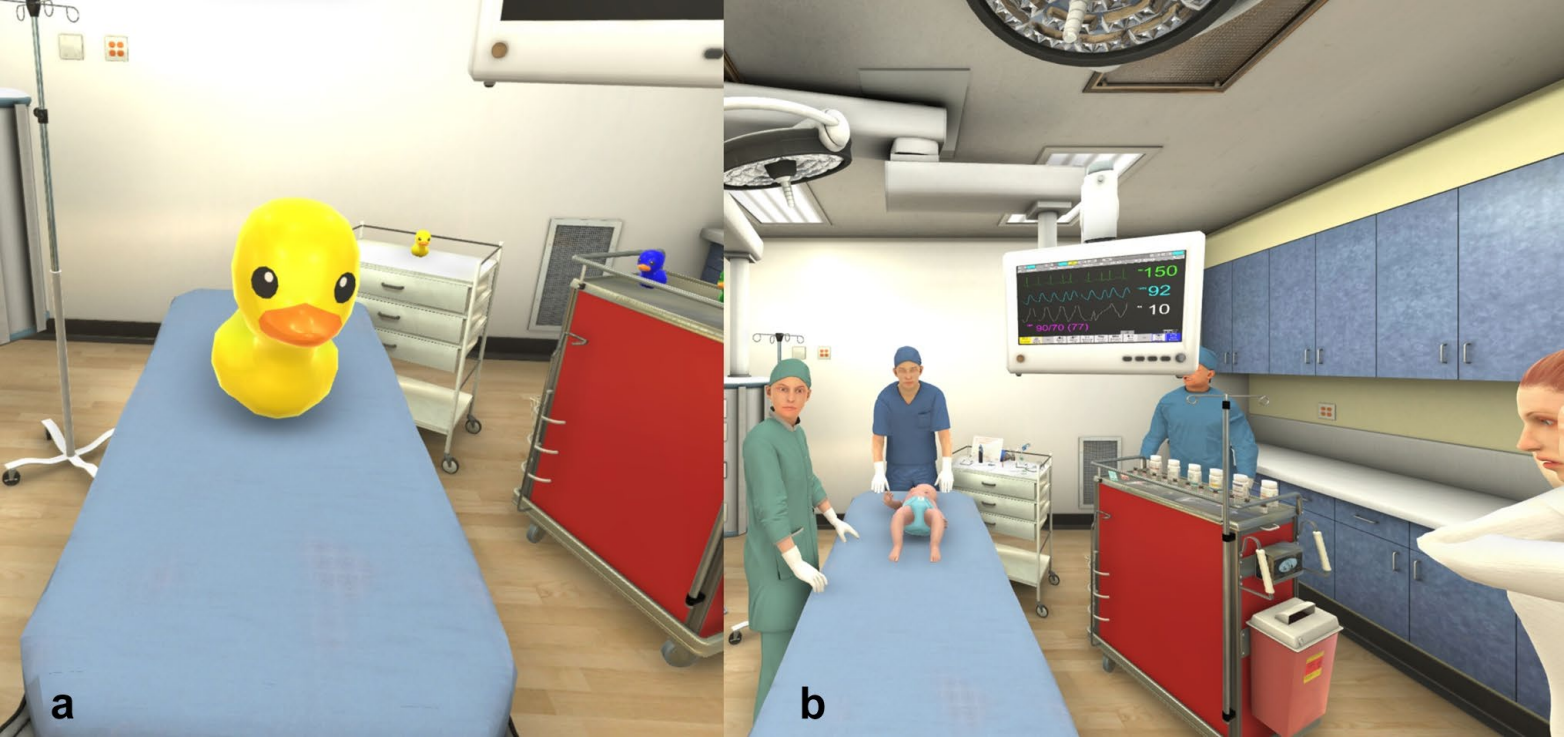
**a **The rubber ducks used in the tutorial to help the students to familiarize with the interaction with the objects in the software; **b** screenshot from the point of view of the student during the scenario; in the scene are present (from right to left) the mother of the patient, a nurse, a respiratory therapist, another nurse and, on the bed in the center, the patient

During the scenarios, the student plays the emergency department physician beginning at the foot of the patient’s bed. Patient information is briefed by associated characters including a paramedic; the rest of the scenario requires interaction with other characters, including a nurse and respiratory therapist. A distraught mother is present in the scenario as a potential distraction (Fig. 1b). The student interacts with the characters and various equipment and medications in the room in response to changing patient states. Medication doses or age-based size of equipment are not required in the scenarios.

Nursing students were primed to engage in the VR sessions and play the physician role in the scenarios, with the purpose of learning the ABCDE systematic approach to the assessment and management of pediatric emergencies. All students participated in the VR sessions on a voluntary basis, as this was a pilot innovative teaching module.

A facilitator-centered debriefing was done after each scenario, and students could then rerun the scenarios to apply what they had learned.

At the end of the sessions, students completed a Technology Assessment Questionnaire to evaluate the *perceived usefulness* and *perceived ease-of-use *(Supplementary file 1). This questionnaire was developed from an engineering and programming perspective and is used for digital training products by i3 Simulations. Students were also asked to rate their *perceived level of competence* in managing a clinical scenario of pediatric status epilepticus and of pediatric anaphylaxis before and after the VR session on a five-point Likert scale, from “very low” to “very high.”

Overall, 45 students participated in this pilot project, 38 last year medical students and 7 last year nursing students. One felt nauseated and could not complete the VR session.

The median perceived usefulness was 91.7/100 (interquartile range (IQR) 80.6–100), while the median perceived ease-of-use was 77.8/100 (IQR 63.9–88.9). The perceived level of competence increased from 2 (IQR 1–3) to 4 (IQR 3–4) for both scenarios (*p* < 0.001, from Wilcoxon test for paired samples).

We noted that ease-of-use was high, but not as high as the perceived usefulness. When triangulating these findings with student comments, students had more comments about the feasibility and suggestions for improvement. In particular, despite the students’ generational advantage with general technologies, they uniformly requested further efforts to familiarize themselves with the VR hardware and an opportunity to “play around” in the digital VR environment, including with medical equipment and medications, before using them on the “real” patient.

Our work presents some limitations inherent to its pilot design, including the single center location and the small sample size. In addition, we included both medical and nursing students as these pilot sessions aimed to assess the feasibility and usefulness of the VR simulation training across mixed clinical background, so that to gear the introduction and debriefing to the participants’ needs. While we could not analyze nursing students’ responses separately, as the questionnaires were completed anonymously using a personal identifier code unknown to the research team, we did not find outliers in our analyses.

The feedback and information from this pilot group of students on feasibility were well-received, and further work went into closing the gap between their unfamiliarity with VR hardware in general and lack of familiarity with the software. Several iterative meetings among all authors, including the initial programmers of the software, yielded two implementable changes.

First, we have learned that despite a generational advantage for technologies among millennials and younger medical students, an assumption that they will navigate VR hardware in a medical training setting should not be made. We discussed as a team whether an in-person hardware demonstration could be enhanced by a video-based hardware demonstration available to participants for watching before the VR session. We have thus developed a video-based demonstration for students to watch at their own leisure prior to a VR session. This approach is currently being evaluated in a new VR project where we expect to find an overall higher participants’ rating of perceived ease-of-use.

Second, the programmers agreed on revamping the tutorial. The initial resuscitationVR tutorial was made to eliminate the possibility that the student will be primed about the topic of the case scenario for the purposes of prior research studies [9, 11]. Ultimately, for VR implementation on PEM training among students, we felt that it was important for students to take the time to explore their digital surroundings just like they would explore their real surroundings on their first day of a clinical rotation, including familiarity with equipment locations and medication availability. Therefore, we introduced a “sandbox” tutorial in which students were free to use and interact with all of the available equipment, medications, and virtual patient outside of a scripted scenario to gain contextual understanding of how the scenario functioned.

Finally, we demonstrate a successful collaboration between industry and an academic teaching center. While contractual relationships between industry and academia are not new, this type of pilot feasibility trial in its intended context provides valuable needs assessment data not just for programming changes but for implementation changes as a whole for curricular development.

### Supplementary Information

Below is the link to the electronic supplementary material.

Supplementary file1 (PDF 158 KB)

## Data Availability

Data will be made available upon request.
