# Supplementary material for: SIMPEDVR: using VR in teaching pediatric emergencies to undergraduate students—a pilot study
Source: Eur J Pediatr. 2023 Oct 16;183(1):499–502. doi: 10.1007/s00431-023-05254-z (PMC10858116; doi:10.1007/s00431-023-05254-z)
Supplement: Supplementary file 1 — Supplementary file1 (PDF 158 KB) [file 431_2023_5254_MOESM1_ESM.pdf]

	Extremely Unlikely	Quite Unlikely	Slightly Unlikely	Neither	Slighlty Likely	Quite Likely	Extremely Likely
**1. Using Resuscitation VR would enable me to learn more quickly**							
**2. Using Resuscitation VR would improve my learning performance**							
**3. Using Resuscitation VR would improve my learning engagement**							
**4. Using Resuscitation VR would improve my learning transfer**							
**5. Using Resuscitation VR would make it easier to learn**							
**6. I would find Resuscitation VR useful in my learning**							
**7. Learning to operate Resuscitation VR was easy for me**							
**8. I found it easy to get Resuscitation VR to do what I want it to do**							
**9. My interaction with Resuscitation VR was clear and understandable**							
**10. I found Resuscitation VR to be flexible to interact with**							
**11. It was easy for me to become skillful at using Resuscitation VR**							
**12. I found Resuscitation VR easy to use**							

Questionnaire scoring

Extremely Unlikely = 0, Extremely Likely = 6

Perceived Usefulness (PU) (out of 100) = (Average of Q1-6) x (16.67)

Perceived Ease-of-Use (PEU) (out of 100) = (Average of Q7-12) x (16.67)
